# Disruption of Circadian Transcriptome in Lung by Acute Sleep Deprivation

**DOI:** 10.3389/fgene.2021.664334

**Published:** 2021-03-30

**Authors:** Yuntao Lu, Bing Liu, Junjie Ma, Shuo Yang, Ju Huang

**Affiliations:** ^1^Department of Pulmonary and Critical Care Medicine, Huadong Hospital, Fudan University, Shanghai, China; ^2^Center for Brain Science, Shanghai Children’s Medical Center, Shanghai Jiao Tong University School of Medicine, Shanghai, China; ^3^CAS Key Laboratory of Computational Biology, Shanghai Institute of Nutrition and Health, University of Chinese Academy of Sciences, Chinese Academy of Sciences, Shanghai, China; ^4^Department of Anatomy and Physiology, Shanghai Jiao Tong University School of Medicine, Shanghai, China

**Keywords:** circadian clock, lung, circadian transcriptome, sleep deprivation, energetic cost

## Abstract

Inadequate sleep prevails in modern society and it impairs the circadian transcriptome. However, to what extent acute sleep deprivation (SD) has impact on the circadian rhythms of peripheral tissues is not clear. Here, we show that in mouse lung, a 10-h acute sleep deprivation can alter the circadian expression of approximately 3,000 genes. We found that circadian rhythm disappears in genes related to metabolism and signaling pathways regulating protein phosphorylation after acute sleep deprivation, while the core circadian regulators do not change much in rhythmicity. Importantly, the strong positive correlation between mean expression and amplitude (E-A correlation) of cycling genes has been validated in both control and sleep deprivation conditions, supporting the energetic cost optimization model of circadian gene expression. Thus, we reveal that acute sleep deprivation leads to a profound change in the circadian gene transcription that influences the biological functions in lung.

## Introduction

Lack of sleep is a commonplace in modern society. Inadequate sleep leads to decreased performance and deterioration in health. Sleep gives our body a chance to repair itself, so the lack of sleep can have harmful health effects. Circadian rhythms are tightly related with the cycle of sleep and wakefulness (Medic et al., 2017). Inadequate or irregular sleep can disrupt the circadian rhythms not only in the brain, but also in peripheral tissues (Huang et al., 2011; Moller-Levet et al., 2013; Archer and Oster, 2015). It has bad consequences on the outcome of health and is associated with several medical conditions, including diabetes, heart disease, stroke, high blood pressure, kidney disease, and mood disorders (Goel et al., 2013; Touitou et al., 2017). Particularly, in the pulmonary system, disruption of circadian rhythm accelerates lung cancer due to the enhanced cell proliferation and the metabolic deregulation (Papagiannakopoulos et al., 2016).

The circadian clock in animals orchestrates genome-wide oscillatory gene expression in a roughly 24-h rhythmicity manner. The molecular mechanism of the circadian oscillator as a transcriptional-translational feedback loop has been identified in multiple species including insects and mammals (King and Takahashi, 2000; Young and Kay, 2001; Hurley et al., 2016). In mouse, two transcriptional activators (*CLOCK* and *BMAL1*), together with their inhibitors (*PER1*, *PER2*, *CRY1*, and *CRY2*) consist of a circadian oscillation transcriptional network that bridge the cycling gene expression and the circadian control of organ’s function. Circadian clocks work in a cell-autonomous manner across all major organs and tissues of the body (Schibler, 2005). In the hierarchical organization of circadian gene transcription, the hypothalamic suprachiasmatic nucleus (SCN) acts as a master pacemaker to synchronize or entrain peripheral clocks throughout the body (Husse et al., 2015). Nevertheless, it is thought that proximally 5–20% of genes expressed in any particular tissue including lung undergo circadian oscillations at the mRNA level (Sun et al., 2020).

Understanding the circadian gene transcription regulation bears significant indication of the biology of lung function, especially while we are facing the situations of the prevailing coronavirus pandemic and the increased incidence of lung cancers nowadays. Reports have shown that many people are facing sleep problems [sleep deprivation (SD)] due to the stress and anxiety caused by COVID-19 (Morin and Carrier, 2020). Thus, it is of importance to know how lack of sleep can have effects on the peripheral clocks especially the regulation of circadian gene transcription in lung. In this study, we found that the oscillation pattern of ~3,000 genes has been changed, and the phase of rhythmic mRNA expression in lung was dramatically shifted after a 10-h sleep deprivation. The genes that lost their circadian expression rhythm after acute sleep deprivation are enriched in biological pathways including metabolism and protein phosphorylation, while the genes that obtained rhythmicity after sleep deprivation are mainly related to cell morphogenesis. Here, we demonstrate that sleep debt effects gene expression rhythmicity with significant implication for its effect on health.

## Materials and Methods

### Animals

All experiments were performed in accordance with the Institutional Animal Care and Use Committee at Shanghai Jiaotong University School of Medicine. Six- to eight-week-old male C57BL/6 mice housed in a 12 h-light:12 h-darkness (light on at 7:00 AM and light off at 19:00 PM) schedule with *ad libitum* water and mouse chow supply.

### Sleep Deprivation

The mice were randomly divided into experimental and control group (12 mice for each group). Animals in the experimental group were subjected to the sleep deprivation instrument, in which animals were kept awake *via* forced locomotion through a slowly rotating drum (40 cm in diameter, 0.4 m per min). Mice had free access to food and water in the drum. All 12 mice in experimental group were placed in the sleep deprivation device for 10 h (6:00 AM–4:00 PM) during their normal resting phase while the control animals were undisturbed. Then fresh lung tissues were obtained at next six circadian time (CT) points with an interval of 4 h (CT4, CT8, CT12, CT16, CT20, and CT24).

### RNA-seq

The lung samples from 24 mice were sent to Beijing Novogene Co., Ltd. (Beijing, China) for RNA sequencing, using Illumina Novaseq 6000. The samples were firstly qualified using 1% agarose gel electrophoresis for possible contamination and degradation. RNA integrity and quantity were finally measured using RNA Nano 6000 Assay Kit of the Bioanalyzer 2100 system (Masotti and Preckel, 2006). RNA libraries were prepared as described previously (Takahashi et al., 2015). Raw reads were tested for quality using FastQC. The resulting reads (FASTQ files) were aligned to the mm10 annotation from UCSC using Hisat2 (Pertea et al., 2016). The average fragments per kilobase of transcripts per million reads (FPKM) value for each gene was calculated separately for each circadian time point of the two conditions.

### Analysis of RNA-seq Data

To identify periodically expressed genes, the list was further filtered based on expressional level, with a cutoff of FPKM > 1 in 80% samples (*n* = 16,505 genes left in the final dataset). After the filterion, MetaCycle, an R package that incorporates ARSER, JTK_CYCLE, and Lomb-Scargle to conveniently evaluate periodicity in time-series data, was performed to identify oscillation genes (Wu et al., 2016). The output result of MetaCycle contained *p* value, period, and revised amplitude (Wu et al., 2016). Circadian transcripts were defined based on the cutoff of adjusted *p* < 0.05.

**Figure 1:**
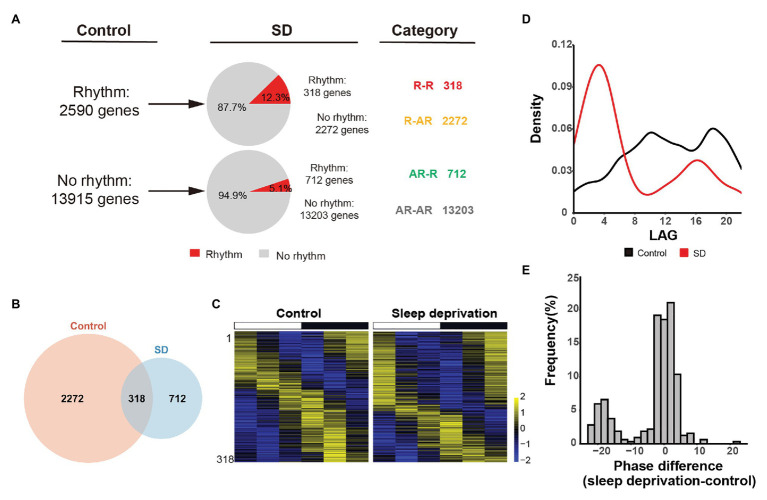
Sleep deprivation alters the circadian gene transcription in lung. **(A)** Quantification of the number of genes that are rhythmically transcribed in the mouse lung. The rhythmic signals are detected by JTK_CYCLE from time-series datasets. Four categories of rhythmically expressed genes were determined by comparing the control and sleep deprivation conditions: rhythmic genes in both conditions (R-R), rhythmic genes only in control condition (R-AR), rhythmic genes only in SD condition (AR-R), and arrhythmic genes in both conditions (AR-AR). **(B)** Area-proportional Venn diagram comparing rhythmic genes identified in the two conditions. **(C)** Heatmap representation of R-R category for the 318 genes that are rhythmically transcribed in the mouse lung. Classification is based on the phase of mRNA oscillations, and each lane corresponds to one gene. High expression is displayed in yellow (*z*-score > 1) while low expression in blue (*z*-score < 1). **(D)** Phase distribution for 3,620 rhythmic genes in two conditions. **(E)** Distribution of the difference between the phase of cycling gene expression in control and SD for the 318 R-R genes.

Frequency histogram of the difference between the phase of the 318 R-R genes in SD and control conditions was performed by ggplot package in R (Figure 1E), in which the values showed in the *x*-axis equaled the phase of SD minus the phase of control. Heatmaps were generated using the pheatmap package in R to display the oscillated genes based on *z*-score (Kolde, 2018). The gene expression levels were normalized by log_2_ (FPKM + 1) in heatmaps. The Venn Diagram Plotter generated by VennDiagram package was used to compare the rhythmic genes under control and SD conditions (Chen and Boutros, 2011). Gene ontology (GO) analysis on circadian disrupted genes was carried out using GO over-representation test in clusterProfiler package (Yu et al., 2012). A value of *p* < 0.05 was used as a cutoff for significantly enriched terms, and the graphs were drawn by R package enrichplot. Scatter plots were conducted based on log transformed amplitude and FPKM value, and the linear regressions were implemented using the “lm” function in R.

### Weighted Gene Co-expression Network Analysis

Weighted gene co-expression network analysis (WGCNA) was performed using WGCNA package (Langfelder and Horvath, 2008). Only genes for which the FPKM value was greater than 1 in 80% samples were used (*n* = 16,505). Pearson’s correlations between each gene pair were calculated to build an adjacency matrix. A soft-threshold power was automatically calculated to achieve approximate scale-free topology (*R*^2^ > 0.85). Then, the topological overlap measure (TOM) and corresponding dissimilarity (1-TOM) was calculated using adjacency matrix. 1-TOM was used as a distance for gene hierarchical cluster, and then DynamicTree Cut algorithm (Langfelder et al., 2008) was used to identify the modules (defined as clusters of highly interconnected genes). We generated 22 modules in control condition. Each module was named following a different color. Gene set enrichment applied for modules was performed using a Fisher’s exact test in R. Kyoto Encyclopedia of Genes and Genomes (KEGG) pathway (Kanehisa and Goto, 2000) analysis and was conducted to identify circadian disrupted genes at the biologically functional level. The Database for Annotation, Visualization, and Integrated Discovery (DAVID; David.abcc.ncifcrf.gov) was used to integrate functional genomic annotations (Dennis et al., 2003). Value of *p* < 0.05 was considered to indicate a statistically significant difference (Huang et al., 2008).

## Results

### Sleep Deprivation Alters the Circadian Gene Transcription Profile in Lung

To assess the effect of acute sleep deprivation on circadian rhythm of gene expression in lung, we deprived sleep from the mice for 10 h in 1 day. The animals in the control group were housed under 12 h: 12 h light/dark cycle, while the animals in the acute sleep deprivation group (SD) were kept awake for 10 h from 6 AM. to 4 PM according to a sleep deprivation paradigm. After 10-h sleep deprivation, we harvested the lung tissue at six time points with 4-h intervals during the following 24 h. Transcriptome analyses were performed by total RNA-sequencing (whole-transcriptome sequencing). The comparison between control and SD conditions was restricted to genes that were sufficiently expressed in both datasets, with a cutoff of FPKM > 1 in at least 80% samples (16,505 genes left in the final dataset).

To identify oscillated gene expression, we performed MetaCycle analysis and found poor overlap exist between control and SD rhythmic gene sets (adjusted *p* < 0.05, Benjamini and Hochberg’s method). We found that in both conditions, the expression of a large group of genes have exhibited robust oscillation signals (Figure 1A), implicating that cycling transcriptome is a basic feature of gene transcription. Specifically, we identified 2,590 rhythmically expressed genes in control group. Only 12.3% of them (318 out of 2,590 genes) manifested maintained rhythmicity in SD group. The rhythmically expressed genes that maintained rhythmicity after sleep deprivation were referred to as “R-R” gene set (Figures 1A,B). The other 87.7% of rhythmically expressed genes in control group (2,272 out of 2,590 genes) failed to keep rhythmicity after acute sleep deprivation. The rhythmically expressed genes that lost rhythmicity after sleep deprivation were referred to as “R-AR” gene set (Figures 1A,B). In contrast, 712 genes gained rhythmicity after sleep restriction. The *de novo* rhythmically expressed genes after sleep restriction were referred to as “AR-R” gene set (Figures 1A,B).

We found that there were 318 genes in R-R data set that kept rhythmicity after acute sleep deprivation (Figure 1C). However, we found that oscillation phase was dramatically altered after sleep deprivation, when we analyzed the phase of circadian transcripts in both control and SD conditions. The peaks at circadian time 10 (CT10) and circadian time 18 (CT18) in control condition coincided with the trough in SD condition, while the peaks at circadian time 14 (CT4) and circadian time 16 (CT16) in the SD condition concurred with the trough of control (Figure 1D), suggesting that most oscillation genes in lung exhibited a time-dependent transcription profile after acute sleep deprivation. Further, we wanted to know whether the genes in R-R data set kept the phase of rhythmicity. We performed the phase difference calculation (phase of sleep deprivation – phase of control) and found that there was a significant phase shift in rhythmicity between the control and SD conditions (Figure 1E).

### Circadian Rhythm Disappears in Genes Related to Metabolism and Signaling Pathway Regulating Protein Phosphorylation After Sleep Deprivation

**Figure 2:**
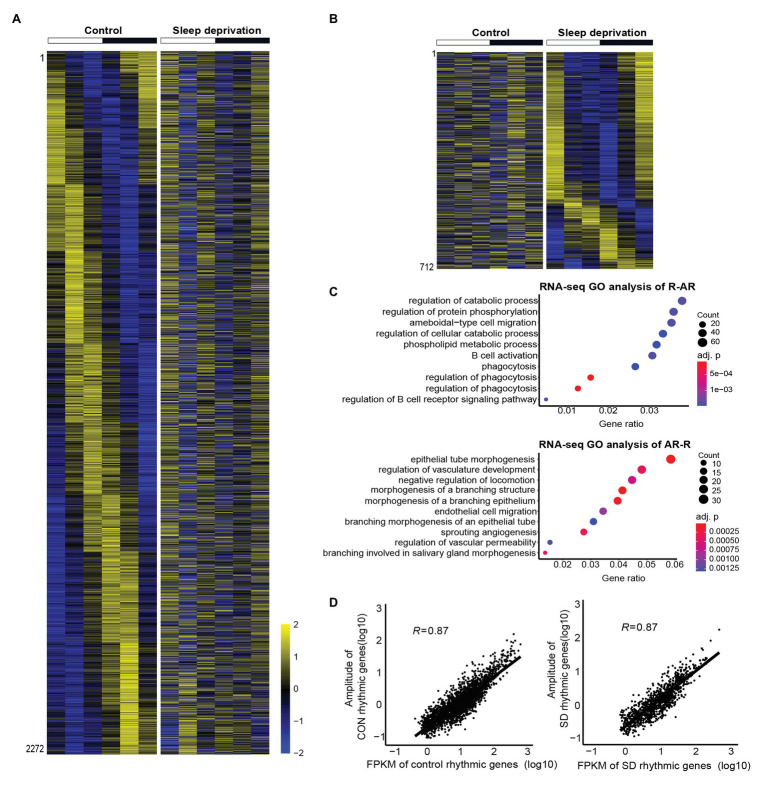
Gene ontology (GO) analyses on the genes that altered rhythmic expression after sleep deprivation. **(A,B)** Heatmap of cyclic mRNA expression in the mouse lung. The majority of rhythmically expressed genes in control condition lost oscillation after sleep deprivation (**A**, *n* = 2,272, R-AR category in Figure 1A). There were many genes that gained circadian oscillation expression after sleep deprivation (**B**, *n* = 712, AR-R category in Figure 1A). High expression is displayed in yellow (*z*-score > 1) and low expression in blue in the heatmaps (*z*-score < 1). **(C)** GO analysis for functional classes in which circadian disrupted genes in the control and SD conditions were enriched. **(D)** Amplitude of rhythmic genes was correlated with mean expression in both control (*n* = 2,590, *R* = 0.87) and SD (*n* = 1,030, *R* = 0.87) conditions.

We found the majority of rhythmically expressed genes (2,272 “R-AR” genes) in control condition lost oscillation in SD condition (Figure 2A). To further understand the functional and biological pathways of those genes, we performed GO analysis on biological processes. We found that those genes were significantly enriched in functional classes, including regulation of catabolic process (*p* = 0.001407), regulation of protein phosphorylation (*p* = 0.001407), ameboidal-type cell migration (*p* = 0.001407), and phospholipid metabolic process (*p* = 0.001479; Figure 2C). In contrast, the 712 “AR-R” genes that gained circadian expression after sleep deprivation were enriched in processes, such as epithelial tube morphogenesis (*p* = 7.63E-06), vasculature development (*p* = 0.000375), morphogenesis of a branching structure (*p* = 6.89E-05), and morphogenesis of a branching epithelium (*p* = 6.89E-05; Figures 2B,C). In addition, we observed a strong positive correlation between mean expression and amplitude (E-A correlation) of periodically expressed genes in both control and SD conditions (*R*^2^ > 0.75, Figure 2D), supporting the energetic optimization models of the transcriptome in the two conditions (Wang et al., 2015; Cheng et al., 2019; Sun et al., 2020). Notably, the E-A correlation in the two conditions was comparable, implicating that a similar level of optimization on the transcriptome was achieved.

### Core Circadian Regulators Maintain Rhythmicity After Sleep Deprivation

**Figure 3:**
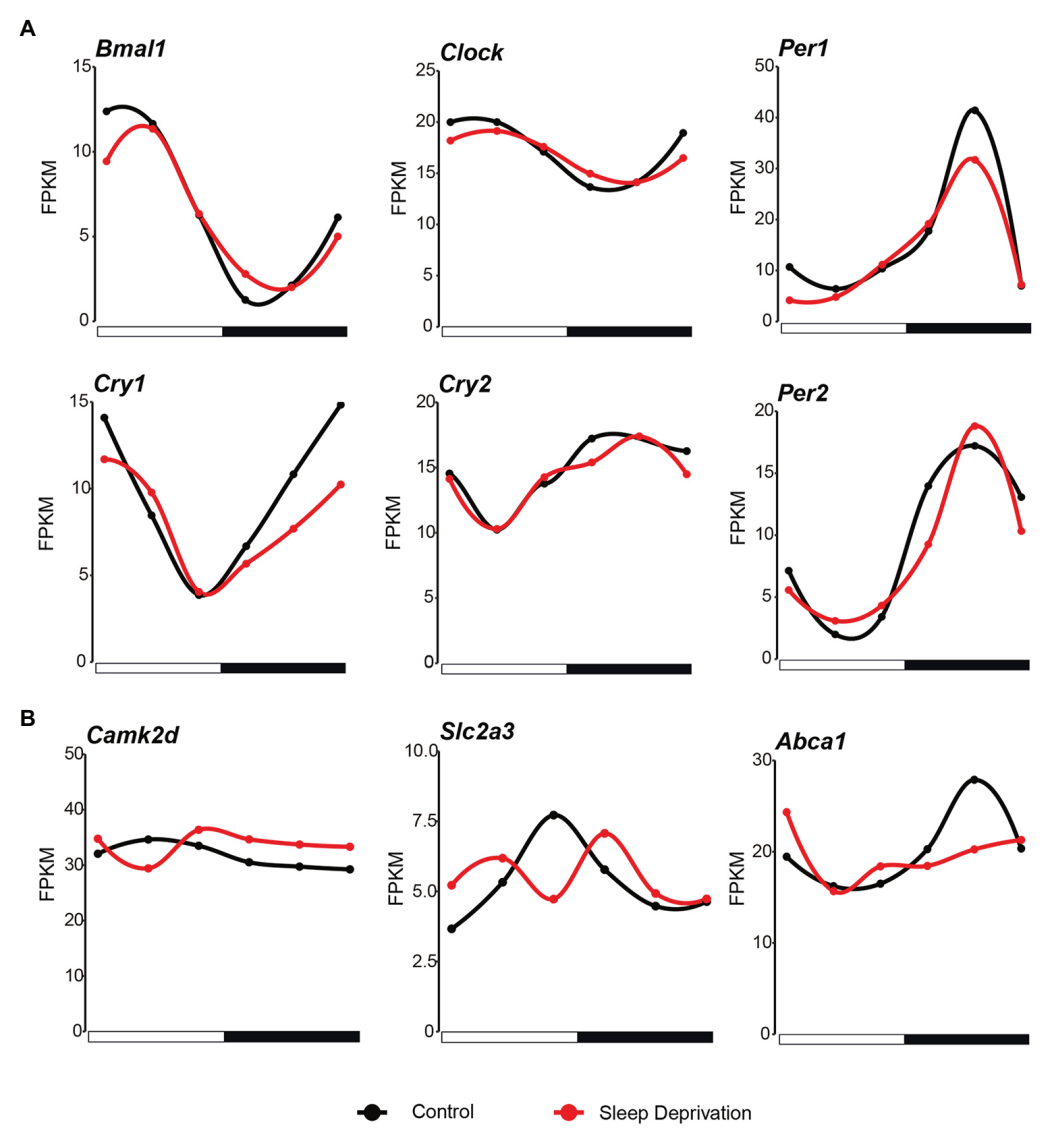
Core circadian regulators maintain rhythmicity after sleep deprivation. **(A)** The circadian expression of core clock genes in the mouse lung during the 24 h in both control (black lines; time points every 4 h starting at CT4) and SD (red lines; time points every 4 h starting at CT4) conditions. **(B)** The mRNA expression of genes that changed the circadian behavior compared with human after sleep deprivation (black lines; time points every 4 h starting at CT4) and SD (red lines; time points every 4 h starting at CT4) conditions.

As more than 2,200 genes lost rhythmicity in their gene expression after a 10-h sleep deprivation, we next examined whether there was a significantly oscillation behavior changes in the core circadian regulatory network. We found that the overall circadian behavior does not change much in the core transcriptional factors. The six core circadian regulators (*Bmal1*, *Clock*, *Per1*, *Per2*, *Cry1*, and *Cry2*) still exhibited strong oscillation signal after acute sleep deprivation (Figure 3A). However, we found that the amplitude of *Per1* and *Cry1* was slightly decreased in SD condition (Figure 3A). The detailed effects of the changing amplitude of core circadian regulators need more sophisticated modeling. Further, compared with the effects of insufficient sleep on human blood samples, it seems that *Camk2d*, which is related to sleep homeostasis, and *SLC2A3* and *ABCA1*, which are related to metabolism, changed their oscillation behavior in SD condition (Figure 3B).

### Large-Scale Co-expressed Gene Networks Are Associated With Period Heterogeneity

**Figure 4:**
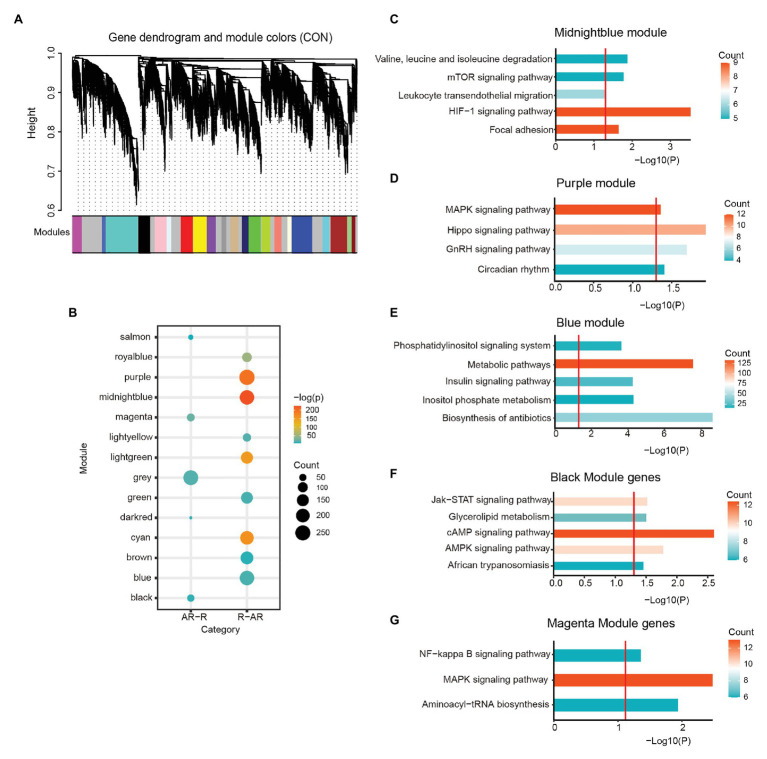
Weighted gene co-expression network analysis (WGCNA) analyses on the circadian disrupted genes. **(A)** Hierarchical cluster analysis was conducted to detect co-expression clusters with corresponding color assignments. Each color represented a module in the constructed gene co-expression network by WGCNA. Twenty-two modules were identified and marked by colors on the horizontal bar. **(B)** Bubble plot showing enrichment of AR-R (*n* = 712) and R-AR (*n* = 2,272) genes in the main modules. Values of *p* were calculated using the Hypergeometric test. **(C–G)** Top ingenuity pathways associated with the midnight blue module **(C)**, the purple module **(D)**, the blue module **(E)**, the black module **(F),** and the magenta module **(G)**. Red threshold lines refer to *p* = 0.05.

Because functionally related genes are usually co-expressed (Heyer et al., 1999), we further characterized the circadian disrupted genes by examining their co-expression patterns. Using WGCNA, we generated 22 modules from 16,505 genes in control condition (Figure 4A). The soft-threshold for network construction was selected as 12 to make a scale-free network (Figure 4A). Several modules exhibited significant enrichment for circadian disrupted genes (Figure 4B). Royal blue, purple, midnight blue, light green, and cyan modules were enriched in R-AR gene set, while salmon, magenta, and black modules were enriched for AR-R gene set (Figure 4B). In this WGCNA analysis, the genes that were not clustered in any module were in grouped as the gray module, so the subsequent analysis was no longer performed on this module.

To further screen the specific biological functions or pathways of each module, we performed KEGG analysis (Figures 4C–G). Within the R-AR-enriched modules, the “midnight blue” module was enriched in several signaling pathways including the Hypoxia-inducible factor 1 (HIF-1) signaling pathway (Figure 4C; *p* = 2.95E-04). This pathway is known to trigger adaptive responses of cells under hypoxic stress through transcriptional activation of hundreds of downstream genes involved in cancer development (Lu et al., 2016). The “purple” module was enriched in the function of circadian rhythms (*p* = 0.039131), in addition to MAPK signaling pathway (*p* = 0.043868), Hippo signaling pathway (*p* = 0.011556), and GnRH signaling pathway (*p* = 0.020401; Figure 4D). The “blue” module was shown to be functional in metabolic-related processes, further confirming the importance of the circadian-disrupted gene networks (Figure 4E). Given that the HIF-1, MAPK, and Hippo signaling pathways all involve biological processes of protein phosphorylation, the KEGG analyses of R-AR-related modules (Figure 4), together with the GO analyses of R-AR gene set (Figure 2), indicated that sleep deprivation impaired signaling pathways regulating protein phosphorylation. Within the AR-R enriched modules, the “black” module was enriched in cAMP signaling pathway (*p* = 0.0025) (Figure 4F). The “magenta” module was enriched in MAPK signaling pathway (*p* = 0.001295), aminoacyl-tRNA biosynthesis (*p* = 0.005513), and NF-kappa B signaling pathway (*p* = 0.026075; Figure 4G). The 712 genes in AR-R gene set were mainly associated with the regulation of intercellular communication, transcription, and protein synthesis, suggesting that they contributed to gene overexpression and cell proliferation. In addition, the 318 genes that maintained rhythmicity after acute sleep deprivation (the R-R gene set) were mainly associated with the regulation of circadian rhythm (Supplementary Figure S2). Taken together, these results suggested that sleep deprivation resulted in profound changes on several biological processes related to adverse health outcomes.

## Discussion

By utilizing a 10-h sleep deprivation paradigm, we found that >2,500 genes displayed periodically expression signal in the normal light-dark condition, while only ~1,000 genes have oscillated gene expression in the sleep deprivation condition. The gain and loss of rhythmicity of gene expression were associated with widely distributed biological functions. The reason why the “AR-R” genes gained rhythmicity might be due to the altered expression of several transcription factors and cofactor after acute sleep deprivation (Supplementary Figure S1). Although the outcome of the circadian regulatory network dramatically changed, the core circadian transcriptional factors maintained their rhythmicity in this short-period sleep disruption. Finally, the circadian oscillation of gene expression in both normal and SD conditions support the energetic cost optimization model of circadian gene expression.

Although chronic sleep restriction has been proved to show profound impacts on transcription regulation in many peripheral tissues including the liver and adipose tissue (Barclay et al., 2012; Husse et al., 2012), acute sleep deprivation has also been demonstrated to raise risk of developing acute sleep loss-associated adverse outcomes in several peripheral tissues such as adipose tissue (Wilms et al., 2019), and skeletal muscle (Lamon et al., 2021). The RNA-seq data from adipose tissue revealed that acute sleep loss could up-regulate oxidative phosphorylation- and ribosome-related signaling pathways (Cedernaes et al., 2018). Lamon et al. (2021) showed that acute sleep deprivation blunted the muscle protein synthesis that affected the muscle protein turnover in skeletal muscle tissue. Taken together, these studies, as well as our current study, indicated that acute sleep deprivation affected gene expression in a tissue-specific manner by targeting different signaling and biological pathways.

Previously, it was reported that only several hundreds of genes have lost rhythmicity signals in insufficient sleep, such as in human blood (Moller-Levet et al., 2013). There are several reasons why a larger number of genes have been detected in our study. Firstly, we have densely sampled the lung for two replicates with an interval of 4 h, and the sequencing depth for each sample is relatively high (approximately 50 million reads *per* sample); secondly, the peripheral tissue (lung) that we focused is the organ that contains one of the highest number of cycling genes among other organs; thirdly, there are less genetic heterogeneity in the laboratory mouse (C57/BL6 strain) samples than in human samples collected before (Moller-Levet et al., 2013).

We found that circadian rhythm disappears in genes related to metabolism and signaling pathway regulating protein phosphorylation after sleep deprivation. Putative mechanisms linking sleep deprivation and metabolic disorders have been uncovered for both animals (Opperhuizen et al., 2015) and humans (Nedeltcheva and Scheer, 2014). Studies have provided convincing evidence that even short-term decreases in sleep quantity or quality can have deleterious effects on glucose regulation, interfere with the secretion of anabolic hormones including growth hormones and prolactin, and alter the amount and timing of catabolic hormones including glucocorticoids and catecholamines (Spiegel et al., 1999; Penev et al., 2005). Also, the signaling pathway of insulin (phosphorylation of Akt) was disturbed along with abnormal glucose control by sleep restriction (Broussard et al., 2012).

Phosphorylation is one of the most important modalities in the regulation of protein activity and it is used to control the basic function of cells (Cohen, 2002; Nishi et al., 2014). In the nervous system, the global phosphorylation statuses in neurons appear to be controlled by sleep-wake cycles (Wang et al., 2018; Bruning et al., 2019). It is thought that protein phosphorylation and sleep-wake cycles have bidirectional mutual effects on each other. CaMKIIα/CaMKIIβ, SIK3 and ERK1/ERK2 have been identified as sleep-promoting kinases (Ode and Ueda, 2020). Phosphoproteomic studies indicated that SIK3 appears to induce a comparable phosphoproteomic profile to that caused by sleep deprivation, and the dynamics of CaMKII activation correlate well with the expected accumulation of sleep need. These previous studies support our major finding that circadian rhythm disappears in genes related to metabolism and signaling pathway regulating protein phosphorylation after sleep deprivation.

The disappearance of a large number of oscillated genes in lung after a short insufficient sleep has many biomedical implications, especially in the period of COVID-19 pandemic. Patients who have been infected by COVID-19 may face sleep problems due to anxiety and symptoms, such as fever, headache, and difficulty breathing. The short of sleep may influence the circadian regulation of lung itself, aggravating the symptoms of the infection. Thus, COVID-19 patient may be highly advised to have high quality sleep, especially during the infection and recovery period.

Taken together, in this research, we revealed that the plasticity of lung transcriptome was higher than previous thought. A short-term sleep disruption may have a strong effect on the circadian gene expression. Further studies may focus on the consequences of the dramatic expression changes, especially on the direction of the connection between transcriptome plasticity and lung cancer etiology.

## Data Availability Statement

The data files for RNA-seq reported in this article have been deposited in the Gene Expression Omnibus database under GSE166335.

## Ethics Statement

The animal study was reviewed and approved by Institutional Animal Care and Use Committee at Shanghai Jiaotong University School of Medicine.

## Author Contributions

JH and YL designed and supervised the project. BL and JM performed bioinformatics analyses. JH and BL wrote the article. All authors contributed to the article and approved the submitted version.

### Conflict of Interest

The authors declare that the research was conducted in the absence of any commercial or financial relationships that could be construed as a potential conflict of interest.

The handling editor declared a shared affiliation with several of the authors, YZ and YL, at time of the review.

## Supplementary Material

Supplementary Figure S1 Transcription factors gain rhythmicity after sleep deprivation. The expression of some transcription factors in the mouse lung during the 24 h in both control (black lines; time points every 4 h starting at CT4) and sleep deprivation (red lines; time points every 4 h starting at CT4) conditions.

Supplementary Figure S2 KEGG analyses on the genes that maintained rhythmicity after sleep deprivation. Top ingenuity pathways associated with the R-R gene set. Red threshold lines refer to *p* = 0.05.
