# Supplementary figures and images for: Disruption of Circadian Transcriptome in Lung by Acute Sleep Deprivation
Source: Front Genet. 2021 Mar 30;12:664334. doi: 10.3389/fgene.2021.664334 (PMC8042274; doi:10.3389/fgene.2021.664334)

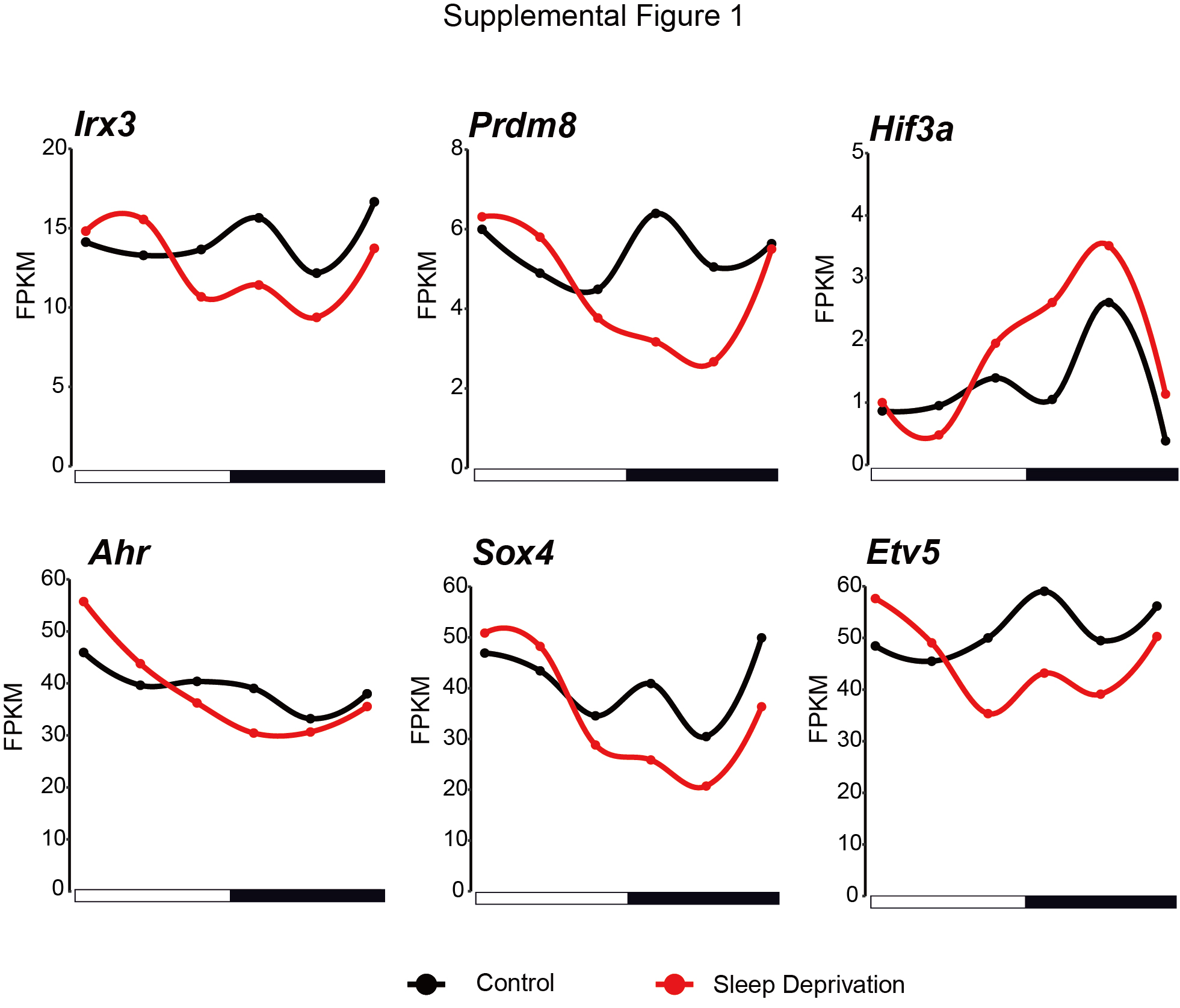

Supplement: Supplementary Figure S1 — Transcription factors gain rhythmicity after sleep deprivation. The expression of some transcription factors in the mouse lung during the 24 h in both control (black lines; time points every 4 h starting at CT4) and sleep deprivation (red lines; time points every 4 h starting at CT4) conditions. [file Image_1.jpeg]

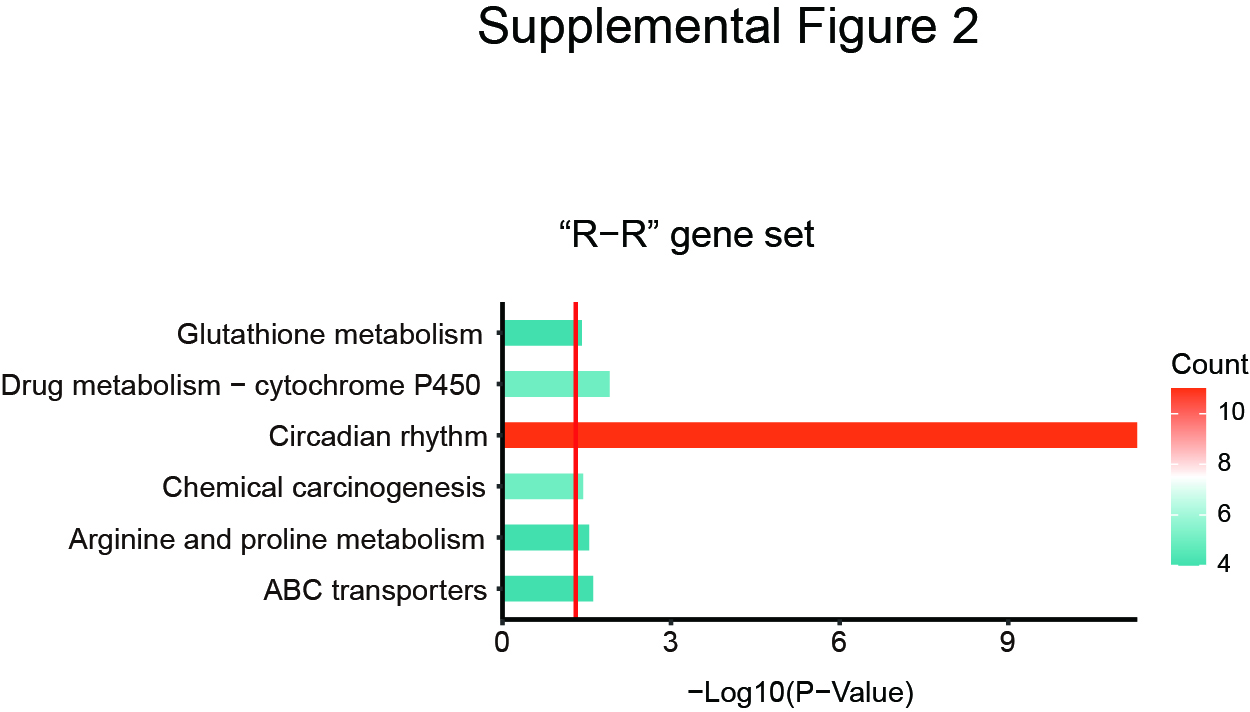

Supplement: Supplementary Figure S2 — KEGG analyses on the genes that maintained rhythmicity after sleep deprivation. Top ingenuity pathways associated with the R-R gene set. Red threshold lines refer to p = 0.05. [file Image_2.jpeg]
